# The Association of the Number of Informal Caregiving Roles with Depression: Moderation of Self-Esteem

**DOI:** 10.1093/geroni/igaf122.4210

**Published:** 2025-12-31

**Authors:** Sehyun Baek, Ernest Gonzales, Shicheng Xu

**Affiliations:** New York University, New York, New York, United States; New York University, New York, New York, United States; New York University, New York, New York, United States

## Abstract

Demographic changes, a decline in the ratio of potential caregivers to the number of adults who need care, highlight the growing prevalence of multiple caregiving roles (e.g., double-decker sandwich caregiving). However, little is known about the extent to which the number of caregiving roles is associated with depression. We examined the association between the number of caregiving roles (no role, 1 caregiving role, and 2+ caregiving roles), derived from caregiving for grandchildren, parents with Activities of Daily Livings (ADLs), parents with Instrumental Activities of Daily Livings (IADLs), spouse with ADLs, and spouse with IADLs) and depression, and whether self-esteem moderates this association. We utilized the data from the 2022 Health and Retirement Study (HRS) and RAND HRS (n = 2,259), guided by the Stress Process Model that posits self-esteem as a resource in the association of caregiving on depression. Results indicated that having 1 caregiving role (b = 1.50, p < .01) and 2+ caregiving roles (b = 2.37, p < .01) were significantly associated with a higher level of depression compared to non-caregivers. Self-esteem moderated the associations: higher self-esteem buffered the impact of caregiving roles on depression (1 role × self-esteem: b = −0.043, p < .01; 2+ roles × self-esteem: b = −0.065, p < .01). Findings underscore the importance of considering the heterogeneity of caregiving roles. For instance, caregiver training and support groups to enhance self-esteem, the National Family Caregiver Support Program should be further expanded, with attention to older adults who shoulder multiple caregiving responsibilities.

